# Self-Rotation of Electrothermally Responsive Liquid Crystal Elastomer-Based Turntable in Steady-State Circuits

**DOI:** 10.3390/polym15234598

**Published:** 2023-12-01

**Authors:** Zongsong Yuan, Junxiu Liu, Guqian Qian, Yuntong Dai, Kai Li

**Affiliations:** 1 College of Civil Engineering, Anhui Jianzhu University, Hefei 230601, China; ys2523578175@163.com (Z.Y.); qianguqian1215@163.com (G.Q.); daiytmechanics@ahjzu.edu.cn (Y.D.); 2 Anhui Province Key Laboratory of Building Structure and Underground Engineering, Anhui Jianzhu University, Hefei 230601, China

**Keywords:** liquid crystal elastomers, self-excited motion, rotation, electrothermally responsive, rope

## Abstract

Self-excited motions, characterized by their ability to harness energy from a consistent environment and self-regulate, exhibit significant potential in micro-devices, autonomous robotics, sensor technology, and energy generation. This study introduces an innovative turntable system based on an electrothermally responsive liquid crystal elastomer (LCE). This system facilitates self-rotation within a steady-state circuit. Employing an electrothermal LCE model, we have modeled and numerically analyzed the nonlinear dynamics of an LCE-rope within steady-state circuits, utilizing the four-order Runge–Kutta method for calculations. The numerical results reveal the emergence of two distinct motion patterns in the turntable system under steady-state conditions: a self-rotation pattern and a static pattern. The self-rotation is initiated when the system’s absorbed energy surpasses the energy lost due to damping effects. Furthermore, this paper delves into the critical conditions necessary for initiating self-rotation and examines the influence of various key dimensionless parameters on the system’s rotation amplitude and frequency. These parameters include gravitational acceleration, the initial position of the mass ball, elastic stiffness of the LCE and spring, limiting temperature, heating zone angle, thermal shrinkage coefficient, and damping factor. Our computational findings establish that these parameters exert a modulatory impact on the rotation amplitude and period. This research enhances the understanding of self-excited motions and offers promising avenues for applications in energy harvesting, monitoring, soft robotics, medical devices, and micro- and nano-devices.

## 1. Introduction

Self-excited motion is characterized by a system’s generation of periodic motion in response to a uniform external stimulus, as documented in various studies [1,2,3,4,5]. This phenomenon significantly diminishes the need for complex system control, as it relies on a constant, rather than a periodic, external stimulus for its periodic motion [6,7]. A key attribute of self-excited motion is its capacity to actively assimilate energy from a stable external environment to sustain its periodic activity. The system’s intrinsic properties, including amplitude and period of the self-excited motion, contribute to its enhanced robustness [8]. Notably, self-excited motion is inherently passive and operates without external control. This attribute facilitates more streamlined system design, fostering intelligence, automation, and resource efficiency, thereby enhancing overall system efficiency [9,10]. The versatile nature of self-excited motion extends its applicability across diverse fields such as energy control [11], autonomous robotics [12,13,14], micro-nano devices [15], and medical technology [16,17,18].

During self-excited motion, energy is dissipated due to damping effects in the system. In order to maintain this self-excited motion, it is usually necessary to provide energy compensation by means of a nonlinear feedback mechanism, which is used to counteract the energy loss of system damping [19,20,21]. For example, energy replenishment can be realized through a self-shading mechanism [22], a mechanism of chemical reaction coupled with large deformation [23], and the coupled motion mechanisms of air expansion and liquid column formation [24,25]. These nonlinear feedback mechanisms are crucial in maintaining the stability of self-excited motion. There has been a notable increase in the reports on self-excited motion systems constructed from active materials, including hydrogels [26,27], dielectric elastomers, ionogels [23], liquid crystal elastomers (LCEs) [28,29,30,31,32], and temperature-sensitive polymers [33,34,35,36]. Meanwhile, numerous efforts have been brought into proposing and constructing a variety of self-excited motion patterns using these active materials, including bending [33,34,35], twisting [36,37], stretching and contracting [38], rolling [22], oscillating [39,40], jumping [41], rotating [42], and even achieving synchronized motions of multiple coupled self-resonators [43].

LCE is a type of electrothermally responsive material that consists of rod-like meso-crystalline monomers with main or side chains of flexible cross-linked polymers to combine rubbery elasticity with liquid crystal anisotropy. When encountering external stimuli such as electricity, heat, light, and magnetism, liquid crystal monomer molecules will rotate or go through phase transitions, modifying their structures and thereby resulting in macroscopic deformation [44,45,46,47,48,49]. Compared to other types of active materials like temperature-sensitive gels, moisture-sensitive gels, pneumatic artificial muscles, and polyelectrolyte gels, LCE is able to achieve self-excited motion and exhibits superior responsiveness and controllability, bringing innovative solutions to relevant applications. The advantageous properties of the LCE material make these LCE-based self-excited motion systems highly stable and reliable, and they can be driven and controlled in a wireless and non-contact manner. As a consequence, the LCE-based self-excited motion systems are of great interest, with a wide range of potential applications in energy control [50,51], autonomous robotics [52,53], micro- and nano-devices [15], and medical devices.

Recent advancements have led to a notable increase in the prevalence of self-excited motion systems utilizing LCE materials [54]. The majority of these systems operate primarily through mechanisms like direct ambient heating or photothermal and photochemical effects [11,24,28,31,55,56,57,58,59,60,61,62,63,64]. Despite this progress, the variety of self-excited motion patterns remains limited, constraining their application potential in active motor systems. Electronic actuation, particularly in practical applications, offers significant advantages in terms of system control and integration. Recent research has shown success in integrating scalable resistive heaters within LCEs, enabling system operation through controlled electrical potentials [49]. This innovation broadens the scope for both control and application of self-excited motion systems. The present study introduces a novel LCE-based turntable system, designed for self-rotation within a steady-state circuit. It explores both the dynamic rotation behavior and the impact of key system parameters. This investigation lays the groundwork for designing more efficient and responsive control systems.

This paper is organized in the following manner. Firstly, in Section 2, we model the nonlinear dynamics of the turntable system based on an electrothermally responsive LCE in a steady-state circuit, and derive the corresponding control equations. In Section 3, we numerically calculate the rotation of the system using the four-order Runge–Kutta method. We investigate the two motion patterns of the system and the corresponding mechanism. Then, in Section 4, we explore the triggering conditions of the system rotation and the influence of the system parameters on the rotation frequency, and we analyze the rotation behavior under different parameter settings as well. Finally, some conclusions and outlooks are given.

## 2. Model and Formulation

This section delineates the construction of an LCE-based turntable system within a steady-state circuit, accompanied by the development of a theoretical model predicated on the dynamic behavior of LCEs. Key areas of focus encompass the dynamics of the LCE-based turntable system, the progression of molecules within the LCE material, the approach to resolving differential control equations with variable coefficients, and the dimensionless quantification of system parameters.

### 2.1. Dynamics of an LCE-Based Turntable System

**Figure 1:**
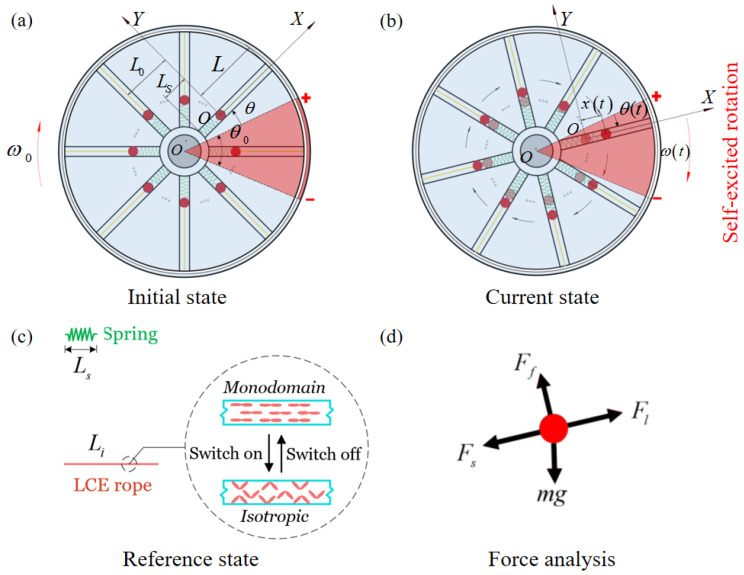
Schematic of an LCE-based turntable system in steady-state circuits, including two layers of discs and *n* motion tubes. Each motion tube contains an electrothermally responsive LCE-rope, a spring of conventional material, and a mass ball. The red region represents the heating zone and the blue region is the heat insulation zone. (**a**) Initial state; (**b**) current state; (**c**) reference state; (**d**) force analysis of the mass ball, which is subjected to gravity mg, damping force $F_{f}$, spring force $F_{s}$ of the spring, and tensile force $F_{l}$ of the LCE-rope. The mass ball keeps entering the heating zone, and the mass ball entering the heating zone will move outward under the tension of the LCE-rope, thus generating a torque difference and sustaining the system in periodic motion.

As shown in Figure 1, this model is an electrothermally responsive LCE-based turntable system rotating around a point $O^{\prime }$. The turntable as a whole comprises two layers of discs, with the upper disc being the driving layer, and the discs are equipped with n motion tubes, each of which contains a small mass ball of mass m, a spring of initial length $L_{s}$, and an LCE-rope of initial length $L_{i}$. The mass ball is placed in the middle of the tube, the inner side connects the mass ball to the turntable with a spring, and the outer side connects the mass ball to the turntable with an LCE-rope. The initial position of the mass ball is at a distance of $L_{0}$ from the center $O^{\prime }$ of the turntable. The lower disc is the electric heating layer, the blue region is the heat insulation zone, whose temperature is always stable and unchanged, and the red region denotes the heating zone, the angle of which is $\theta_{0}$. The resistance wires are evenly laid in the heating zone and connected to the steady-state circuit. The heat generated due to the thermal effect of the electric current raises the temperature of the entire heating zone. The upper boundary of the heating zone is the starting position of the turntable, and the angle between the first mass ball and the starting position is $\theta_{i}$. The current length of LCE-rope is denoted as $L\left(t\right)$. A right-angled coordinate system Oxy is established with the initial position of the mass ball as the coordinate origin, and the turntable is given an initial angular velocity $w_{0}$ of clockwise rotation.

Figure 1c illustrates the modeling of electrothermally responsive LCE-rope in a steady-state circuit. This LCE material is composed of anisotropic rod-shaped liquid crystal molecules and stretchable long-chain polymers that are capable of achieving large and reversible actuation strains, which are mainly caused by the phase transition from the nematic to isotropic phase and the reorientation of the liquid crystal mesogens [28,64]. The monodomain LCE-rope is produced by initially loosely cross-linking the mixture within a circular mold, followed by fully cross-linking through uniaxial stretching and exposure to ultraviolet light. The LCE-rope is in a monodomain state in which the liquid crystal mesogens are arranged axially. When the circuit is connected, the temperature rises, and the LCE-rope will shrink along its axial direction. When the circuit is disconnected, the temperature decreases, and the rope is able to fully recover to its initial length. In the disconnected state, the length of the LCE-rope is $L_{i}$, which serves as a reference state.

During the rotation of the turntable, the following control equation can be derived from the momentum moment theorem:

(1) $$\sum_{i=1}^{n}\left({\dot{J}}_{i}\dot{\theta }+J_{i}\ddot{\theta }\right)=\sum_{i=1}^{n}\left\{mg\cos\left[\frac{\theta_{0}}{2}-\theta -\frac{2\pi }{n}\left(i-1\right)\right]x_{i}-\beta \dot{\theta }(t){\left(L_{0}+x_{i}\right)}^{2}\right\}$$

where $J_{i}=m{\left(L_{0}+x_{i}\right)}^{2}$, β is the damping factor, g is the gravitational acceleration, $x_{i}\left(t\right)$ is the displacement of the ith mass ball, $\dot{\theta }=\frac{d\theta \left(\bar{t}\right)}{d\bar{t}}$ and $\ddot{\theta }=\frac{d^{2}\theta \left(\bar{t}\right)}{d{\bar{t}}^{2}}$ represent the rotation angular velocity and acceleration of the system, respectively.

As described in Figure 1b, when the motion tube enters the heating zone, the LCE-rope experiences an electrothermally driven shrinkage and pulls the mass ball towards the outside of the turntable, and the displacement moved by the mass ball is recorded as $x\left(t\right)$, which generates a gravitational moment difference to promote the rotation of the system. The mass ball enters the heating zone and is subjected to four forces, i.e., the spring force $F_{s}$ of the spring, the tension $F_{l}$ of the LCE-rope, the gravity mg of the ball itself, and the damping force $F_{f}$. It is assumed that the gravity is negligible relative to the elastic force. Since the ball is in equilibrium at any moment in the x-axis direction, we can obtain its equilibrium equation as

(2) $$F_{s}=F_{l}$$

where $\theta \left(t\right)$ is the rotation angle. In accordance with Hooke’s law, we can further simplify its equilibrium equation as

(3) $$F_{s}=k_{s}x\left(t\right),\;F_{l}=k_{l}\left[x\left(t\right)+L_{i}\varepsilon_{T}\left(t\right)\right]$$

where $k_{s}$ refers to the elastic stiffness of the spring, $k_{l}$ refers to the elastic stiffness of the LCE-rope, and $\varepsilon_{T}\left(t\right)$ is the thermally driven contraction strain of the LCE-rope.

Inserting Equation (3) into Equation (2) leads to

(4) $$x_{i}\left(t\right)=-\frac{k_{l}\varepsilon_{T}\left(t\right)L_{i}L_{s}}{k_{s}L_{i}+k_{l}L_{s}}(i=1,2,3,\ldots ,n)$$

where $x_{i}\left(t\right)$ is the displacement of the ith mass ball.

The thermally driven contraction strain of the LCE-rope can be calculated as

(5) $$\varepsilon_{T}\left(t\right)=\alpha T$$

where T is the temperature difference between the LCE-rope and the environment, and α is the coefficient of thermal contraction of the LCE-rope.

Based on Equations (2)–(4), Equation (1) can be simplified as

(6) $$\begin{array}{l} \ddot{\theta }=\frac{\sum_{i=1}^{n}\left\{mg\cos\left[\frac{\theta_{0}}{2}-\theta -\frac{2\pi }{n}\left(i-1\right)\right]x_{i}-\beta \dot{\theta }(t){\left(L_{0}+x_{i}\right)}^{2}-{\dot{J}}_{i}\dot{\theta }\right\}}{\sum_{i=1}^{n}J_{i}} \end{array}$$

### 2.2. Temperature Field in LCE

In this section, the thermal dynamics of an electrothermally responsive system, encompassing both heated and unheated zones, are detailed. The assumption is made that heat exchange within the heating zone occurs rapidly, resulting in a uniform temperature across the LCE-rope. The presence of electric current induces a thermal effect, leading to heat generation in the resistance wire of the turntable’s heating zone upon activation. The quantity of heat produced per second due to the electrothermal effect is represented by the parameter q. The LCE-rope is capable of thermal interaction with the turntable’s heating zone, and it is presumed that the density of heat flow is linearly correlated to the temperature differential T between the LCE-rope and its surrounding environment. Within the heating zone, this temperature difference T is defined as per equation [42].

(7) $$\dot{T}=\frac{q-KT}{\rho_{c}}$$

(8) $$T=T_{L}-T_{e}$$

where $\rho_{c}$ refers to the specific heat capacity, K indicates the heat transfer coefficient, $T_{L}$ is the LCE-rope temperature, and $T_{e}$ is the ambient temperature.

By solving Equation (7), it can be seen that, in the heating zone, the temperature difference T is expressed as

(9) $$T_{n+1}=T_{n}+\frac{t}{\tau }(T_{0}-T_{n})$$

where $T_{0}=\frac{q}{K}$ denotes the limiting temperature difference of the electrothermally responsive LCE-rope in the case of long-time energization, $\tau =\frac{\rho_{c}}{K}$ is the characteristic time of heat exchange between the LCE-rope and the environment, and the larger τ is, the longer the time required for the LCE to reach the limiting temperature difference $T_{0}$.

In the unheated zone, when $T_{0}=0$, the temperature difference T is

(10) $$T_{n+1}=T_{n}-\frac{t}{\tau }T_{n}$$

### 2.3. Nondimensionalization and Solution Method

For convenience, the dimensionless quantities are introduced as follows: ${\bar{x}}_{1}\left(t\right)=\frac{x_{1}\left(t\right)}{L_{i}}$, ${\bar{x}}_{2}\left(t\right)=\frac{x_{2}\left(t\right)}{L_{i}}$, $\bar{g}=\frac{g\tau^{2}}{L_{i}}$, $\bar{\beta }=\frac{\beta \tau }{m}$, $\bar{t}=\frac{t}{\tau }$, ${\bar{L}}_{0}=\frac{L_{0}}{L_{i}}$, ${\bar{L}}_{s}=\frac{L_{s}}{L_{i}}$, $\bar{L}\left(t\right)=\frac{L\left(t\right)}{L_{i}}$, $\bar{T}=\frac{T}{T_{e}}$, ${\bar{T}}_{0}=\frac{T_{0}}{T_{e}}$, ${\bar{k}}_{s}=\frac{k_{s}\tau^{2}}{m}$, and ${\bar{k}}_{l}=\frac{k_{l}\tau^{2}}{m}$.

Re-substituting the above dimensionless parameters into the aforementioned Equations (3) and (6) yields the dimensionless control equations

(11) $${\bar{x}}_{i}\left(\bar{t}\right)=\frac{{\bar{k}}_{l}\varepsilon_{T}\left(t\right)}{{\bar{k}}_{s}+{\bar{k}}_{l}}$$

(12) $$\bar{\ddot{\theta }}=\frac{\sum_{i=1}^{n}\left\{\bar{g}\cos\left[\frac{\theta_{0}}{2}-\theta -\frac{2\pi }{n}\left(i-1\right)\right]{\bar{x}}_{i}-\bar{\beta }\bar{\dot{\theta }}{\left({\bar{x}}_{i}+{\bar{L}}_{0}\right)}^{2}-2\bar{\dot{\theta }}\sum_{i=1}^{n}\left({\bar{x}}_{i}+{\bar{L}}_{0}\right)\frac{{\bar{k}}_{l}\alpha ({\bar{T}}_{0}-\bar{T})}{{\bar{k}}_{s}+{\bar{k}}_{l}}\right\}}{\sum_{i=1}^{n}{\left({\bar{x}}_{i}+{\bar{L}}_{0}\right)}^{2}}$$

where $\bar{\ddot{\theta }}=\frac{d^{2}\theta \left(\bar{t}\right)}{d{\bar{t}}^{2}}$ and $\bar{\dot{\theta }}=\frac{d\theta \left(\bar{t}\right)}{d\bar{t}}$ represent the dimensionless rotation angular acceleration and velocity of the system, respectively.

Since Equation (12) is a differential equation with variable coefficients, it has no analytic solution. In order to solve this differential equation, we utilize the classical fourth-order Runge–Kutta method with the help of Matlab software (R2016b). The fourth-order Runge–Kutta formula involved is as follows

(13) $$\theta \left(\bar{t}+h\right)=\theta \left(\bar{t}\right)+\frac{1}{6}\left(K_{1}+2K_{2}+2K_{3}+K_{4}\right)$$

where $K_{i}(i=1,2,3,4)$ is expressed as

(14) $$\left\{\begin{array}{l} K_{1}=f\left(\bar{t},\theta \right)\\ K_{2}=f\left(\bar{t}+\frac{h}{2},\theta +\frac{h}{2}K_{1}\right)\\ K_{3}=f\left(\bar{t}+\frac{h}{2},\theta +\frac{h}{2}K_{2}\right)\\ K_{4}=f\left(\bar{t}+h,\theta +hK_{3}\right) \end{array}\right.$$

where h is the time step. Ultimately, the dynamic response of the angle and angular velocity for the system rotation over time can be obtained by iterative analysis.

The initial condition of the turntable system can be given as

(15) $$\theta =1.75\pi \;\mathrm{and}\;\bar{w}=1\;\mathrm{at}\;\bar{t}=0.$$

Taking into account the dimensionless parameters including ${\bar{T}}_{0}$, $\theta_{0}$, $\bar{\alpha }$, $\bar{g}$, $\bar{\beta }$, ${\bar{L}}_{0}$, ${\bar{k}}_{l}$, ${\bar{k}}_{s}$, θ, and $\bar{w}$, Equations (3), (4), (6) and (9) can be solved programmatically in Matlab by the four-order Runge–Kutta method. In the calculation, with the position $x_{i-1}$ of the mass ball at the previous moment and the temperature change ${\bar{T}}_{i}$ of the LCE, the current shrinkage strain ${\bar{\varepsilon }}_{i}$ of the LCE can be estimated by Equation (4), which is then combined with Equation (10) to calculate the current position $x_{i}$ of the mass ball. On the basis of ${\bar{\varepsilon }}_{i}$, $x_{i}$, and Equation (12), the current rotation angular velocity of the system can be calculated. When $0\leq \mathrm{mod}\left(\theta_{i},2\pi \right)\leq \theta_{0}$, the mass ball is within the heating zone; otherwise, it is in the heat insulation zone. Repeating the above process, the time course of the rotation angle of the LCE-based turntable system is obtained via iterative calculation.

## 3. Two Motion Patterns and Mechanism of Self-Excited Motion

Utilizing the control equations established earlier and employing numerical analysis, this section is dedicated to examining the dynamic behavior of the system within a steady-state circuit. Initially, the two predominant motion patterns, specifically the static and self-rotation patterns, are introduced. Subsequently, a detailed exploration of the mechanisms driving self-rotation is conducted through a comprehensive parametric analysis.

### 3.1. Two Motion Patterns

To investigate the self-rotation of LCE-based turntable systems, the dimensionless parameters in the theoretical model must first be determined. Based on the available experimental data [11,65,66], the material properties and geometrical parameters of the system, as well as the relevant dimensionless parameters, are listed in detail in Table 1 and Table 2, respectively. Table 1 illustrates the basic material properties and geometrical parameters of the LCE-based turntable system, which are fundamental and indispensable for analyzing the system, while Table 2 shows the associated dimensionless parameters, which are derived based on the fundamental data and the associated dimensionless formulas in Figure 1. These parameters are essential for the study of the effect of self-rotation on the turntable system. In this study, these parameter values will be utilized to investigate the self-rotation characteristics of the LCE-based turntable system in a steady-state circuit.

**Figure 2:**
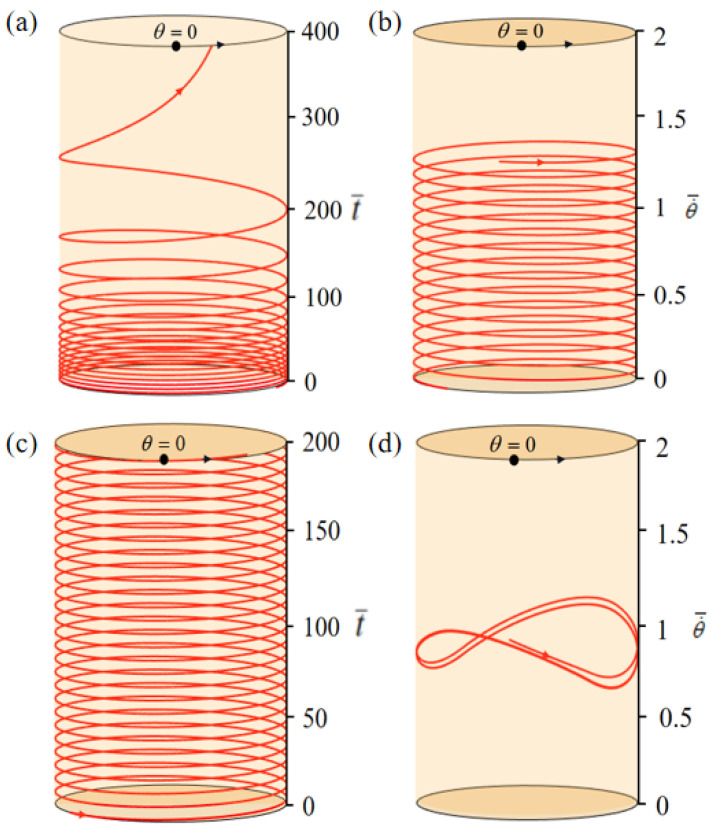
Time histories and phase trajectories of the LCE-based turntable. (**a**,**b**) Static pattern with parameters of ${\bar{T}}_{0}=0$, $\theta_{0}=0.5\pi$, $\bar{\alpha }=0.35$, $\bar{g}=10$, $\bar{\beta }=0.01$, ${\bar{L}}_{0}=1$, ${\bar{k}}_{l}=10$, ${\bar{k}}_{s}=10$, θ=0.25π, and ${\bar{w}}_{0}=1$. (**c**,**d**) Self-rotation with parameters of ${\bar{T}}_{0}=0.35$, $\theta_{0}=0.5\pi$, $\bar{\alpha }=0.35$, $\bar{g}=10$, $\bar{\beta }=0.01$, ${\bar{L}}_{0}=1$, ${\bar{k}}_{l}=10$, ${\bar{k}}_{s}=10$, θ=0.25π, and ${\bar{w}}_{0}=1$. The LCE-based turntable system involves two motion patterns in a steady-state circuit, i.e., static pattern and self-rotation.

From Equations (11) and (12), the phase trajectories and time histories of the system during the rotation in a steady-state circuit can be obtained. In the calculation, we first set ${\bar{T}}_{0}=0$, $\theta_{0}=0.5\pi$, $\bar{\alpha }=0.35$, $\bar{g}=10$, $\bar{\beta }=0.01$, ${\bar{L}}_{0}=1$, ${\bar{k}}_{l}=10$, ${\bar{k}}_{s}=10$, θ=0.25π, and ${\bar{w}}_{0}=1$. With this group of parameters, the turntable begins to rotate with an initial speed of ${\bar{w}}_{0}=1$. And since ${\bar{T}}_{0}=0$ indicates that the circuit is in a power-off state, the LCE-rope does not change after entering the heating zone and the turntable continues to rotate. Due to the air damping, the rotation velocity of the turntable decreases and finally remains stable, which is termed the static pattern, as plotted in Figure 2a,b. When the parameters are set to ${\bar{T}}_{0}=0.35$, $\theta_{0}=0.5\pi$, $\bar{\alpha }=0.35$, $\bar{g}=10$, $\bar{\beta }=0.01$, ${\bar{L}}_{0}=1$, ${\bar{k}}_{l}=10$, ${\bar{k}}_{s}=10$, θ=0.25π, and ${\bar{w}}_{0}=1$, as depicted in Figure 2c,d, the turntable can rotate continuously and eventually develop into a self-rotation, named the self-rotation. Similar to other self-excited motion systems, the LCE-based turntable system demonstrates the capability to execute rotation motion within a steady-state circuit. This phenomenon is primarily due to the external energy input offsetting damping losses, thereby sustaining the self-rotation. Section 3.2 will delve into the intricate mechanisms underlying this self-rotation.

### 3.2. Mechanism of the Self-Excited Motion

**Figure 3:**
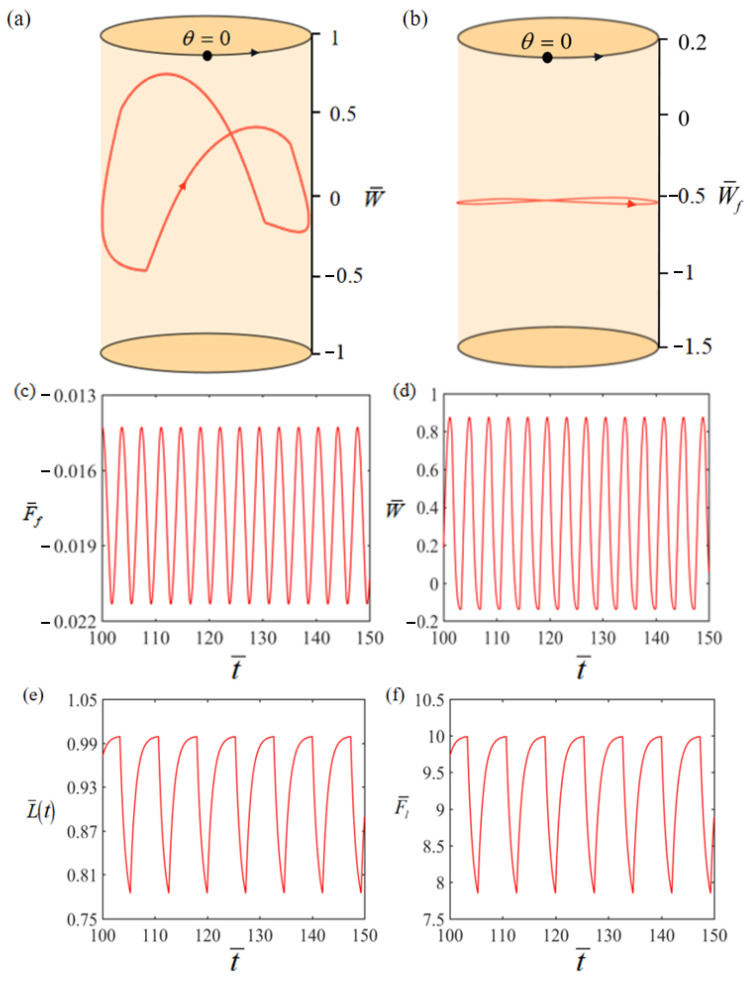
(**a**) Time dependence of the electrothermally driven shrinkage strain in the LCE-rope; (**b**) time dependence of the elastic force; (**c**) time dependence of the damping force; (**d**) time dependence of the driving torque; (**e**) driving torque vs. rotation angle; (**f**) damping torque vs. rotation angle.

**Figure 4:**
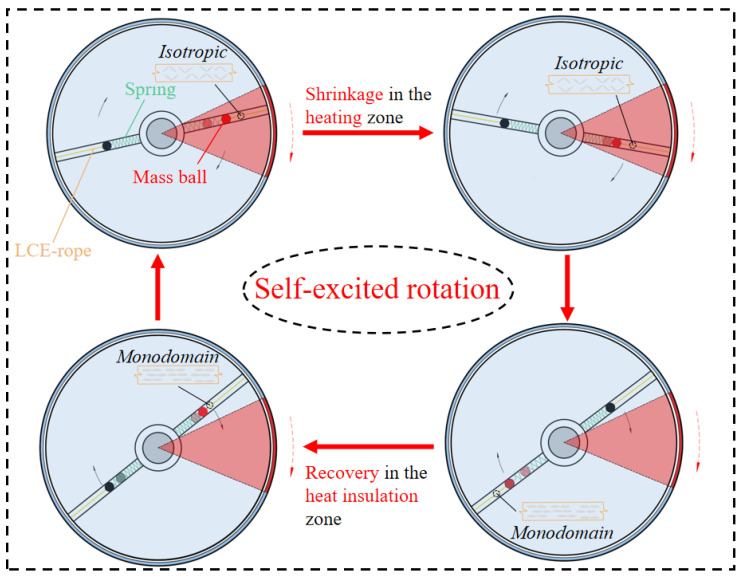
Self-rotation of the turntable system during one cycle. The LCE-rope contracts axially in the heating zone, pulling the mass ball outward, and the electrothermally driven contraction recovers axially in the heat insulation zone. In the steady-state circuit, the LCE-based turntable system exhibits continuous periodic rotation due to the periodic variation of the electrothermally driven contraction of the LCE-rope. The red shaded region indicates the heating zone and the blue shaded region indicates the heat insulation zone.

Figure 3 displays the evolution of several key parameters related to the self-rotation depicted in Figure 2c,d in order to facilitate the exploration of the self-rotation mechanism. Figure 4 illustrates the self-rotation of the LCE-based turntable system during one cycle. The time dependence of the electrothermally driven shrinkage strain in the LCE material during the self-rotation is plotted in Figure 3a, showing a periodic variation with time. The time dependences of the elastic force ${\bar{F}}_{l}$ and the damping force ${\bar{F}}_{f}$ on the mass ball are plotted in Figure 3b,c, respectively, both of which vary periodically with time. In Figure 3d, the driving torque is plotted versus time, which gradually increases in the heating zone and decreases in the heat insulation zone. Figure 3e,f reflect the relationship between driving torque, damping torque, and rotation angle, respectively. The areas enclosed by the two curves represent the net work done by the driving torque and the work consumed by the system damping during one cycle, both of which are 0.51. It is the positive net work done by the driving torque that compensates for the energy dissipated by the damping, allowing the system to maintain a continuous and stable motion pattern of self-rotation. 

## 4. Parametric Study

The equations under consideration encompass eight dimensionless parameters: $\bar{g}$, ${\bar{T}}_{0}$, $\bar{\beta }$, ${\bar{L}}_{0}$, $\bar{\alpha }$, ${\bar{k}}_{l}$, ${\bar{k}}_{s}$, and $\theta_{0}$. These parameters play a pivotal role in modulating the self-rotation dynamics of the LCE-based turntable system, as demonstrated in Figure 1. This segment of the study is dedicated to examining the influence of these crucial parameters on the critical conditions, periodicity, and magnitude of self-excited motion in a system equipped with just two mass balls. The objective is to provide insights applicable to various domains such as energy harvesting, power generation, monitoring, soft robotics, medical devices, and micro- and nano-devices. In this context, A and f are employed to denote the dimensionless amplitude and frequency of the system, respectively.

### 4.1. Effect of the Gravitational Acceleration

**Figure 5:**
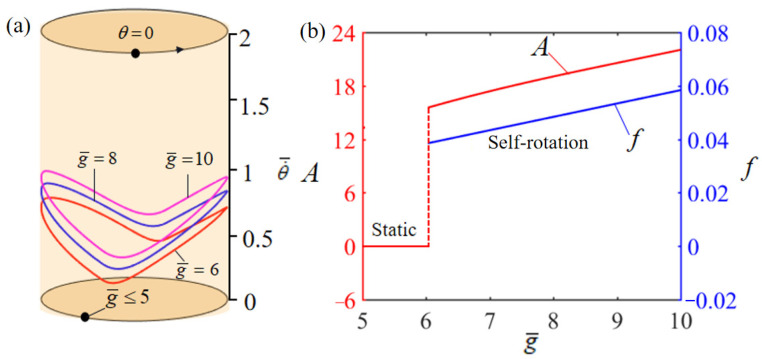
Effect of dimensionless gravitational acceleration on the self-rotation of the LCE-based turntable system, with ${\bar{T}}_{0}=0.35$, $\theta_{0}=0.5\pi$, $\bar{\alpha }=0.35$, $\bar{\beta }=0.01$, ${\bar{L}}_{0}=1$, ${\bar{k}}_{l}=10$, ${\bar{k}}_{s}=10$, θ=0.25π, and ${\bar{w}}_{0}=1$. (**a**) Limit cycle; (**b**) amplitude and frequency.

Figure 5 illustrates the effect of the dimensionless gravitational acceleration $\bar{g}$ on the rotation of the system. In the numerical calculation, we set ${\bar{T}}_{0}=0.35$, $\theta_{0}=0.5\pi$, $\bar{\alpha }=0.35$, $\bar{\beta }=0.01$, ${\bar{L}}_{0}=1$, ${\bar{k}}_{l}=10$, ${\bar{k}}_{s}=10$, θ=0.25π, and ${\bar{w}}_{0}=1$. Figure 5a illustrates the boundary conditions for the rotation behavior of the system under varying gravitational accelerations $\bar{g}$. The analysis reveals a critical gravitational acceleration threshold, specifically $\bar{g}=5$, which initiates rotation motion in the system. At this juncture, $\bar{g}\geq 5$, the system transitions into a rotation mode. This phenomenon is interpretable through the lens of energy balance, considering the interplay between the net energy input and damping losses. Under conditions of low gravitational acceleration, the system can only generate a minimal torque in the heated zone, rendering the net electrothermal energy imparted to the LCE-rope insufficient to offset the energy loss due to damping. In scenarios where the energy input fails to counterbalance the damping losses, the LCE-based turntable system will ultimately cease motion, settling into a static equilibrium in a static state. Figure 5b demonstrates the impact of varying dimensionless gravitational accelerations on both the amplitude of change in the LCE-rope and the system’s rotation frequency. As can be seen in Figure 5, both the amplitude and the rotation frequency increase gradually and more significantly as the gravitational acceleration $\bar{g}$ increases, which is consistent with physical intuition.

### 4.2. Effect of Initial Position of the Mass Ball

**Figure 6:**
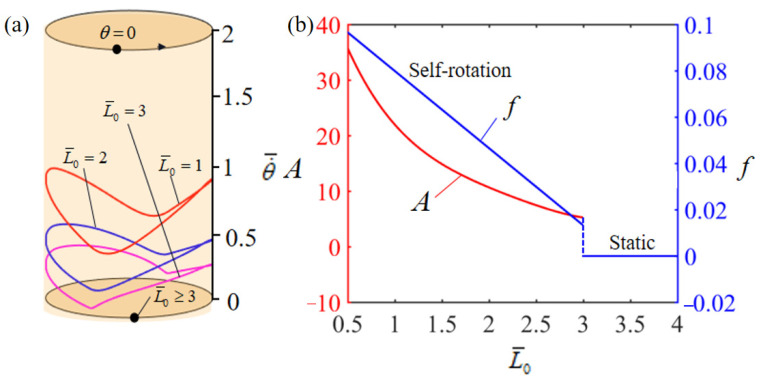
Effect of dimensionless initial position ${\bar{L}}_{0}$ of the mass ball on the self-rotation of the LCE-based turntable system, with ${\bar{T}}_{0}=0.35$, $\theta_{0}=0.5\pi$, $\bar{\alpha }=0.35$, $\bar{\beta }=0.01$, $\bar{g}=10$, ${\bar{k}}_{l}=10$, ${\bar{k}}_{s}=10$, θ=0.25π, and ${\bar{w}}_{0}=1$. (**a**) Limit cycles; (**b**) amplitude and frequency.

Figure 6 describes how the initial position ${\bar{L}}_{0}$ of the mass ball affects the rotation of the system. In the numerical calculation, we set ${\bar{T}}_{0}=0.35$, $\theta_{0}=0.5\pi$, $\bar{\alpha }=0.35$, $\bar{\beta }=0.01$, $\bar{g}=10$, ${\bar{k}}_{l}=10$, ${\bar{k}}_{s}=10$, θ=0.25π, and ${\bar{w}}_{0}=1$. Figure 6a plots the limit cycles of rotation for different initial positions, where a critical initial position of about ${\bar{L}}_{0}=3$ exists for the phase transition between static and self-rotation. When the initial position exceeds or equals the critical value, the energy input to the system from external sources is unable to compensate for the damping loss, which results in the system rotating slower and slower and eventually stopping at the static equilibrium position. When ${\bar{L}}_{0}=1$, ${\bar{L}}_{0}=2$, and ${\bar{L}}_{0}=3$, the self-rotation is triggered with the limit cycles being plotted in Figure 6a. Figure 6b shows how the initial position of the mass ball influences the rotation amplitude and frequency. As the initial distance from the mass ball to the turntable center increases, there are significant decreases in the amplitude and frequency. This result indicates that the increase in initial position is not conducive to the system rotation, but instead makes the system rotate slower and slower, and finally stop at the static equilibrium position. 

### 4.3. Effect of Damping Factor

**Figure 7:**
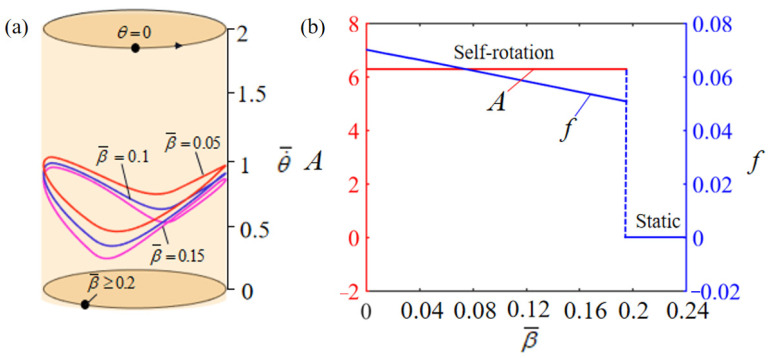
Effect of dimensionless damping factor on self-rotation of the LCE-based turntable system, with ${\bar{T}}_{0}=0.35$, $\theta_{0}=0.5\pi$, $\bar{\alpha }=0.35$, $\bar{g}=10$, ${\bar{L}}_{0}=1$, ${\bar{k}}_{l}=10$, ${\bar{k}}_{s}=10$, θ=0.25π, and ${\bar{w}}_{0}=1$. (**a**) Limit cycles; (**b**) amplitude and frequency.

Figure 7 illustrates the effect of dimensionless damping factor $\bar{\beta }$ on the rotation, with other parameters being set as ${\bar{T}}_{0}=0.35$, $\theta_{0}=0.5\pi$, $\bar{\alpha }=0.35$, $\bar{g}=10$, ${\bar{L}}_{0}=1$, ${\bar{k}}_{l}=10$, ${\bar{k}}_{s}=10$, θ=0.25π, and ${\bar{w}}_{0}=1$. Figure 7a presents the rotation limit cycles corresponding to varying damping factors, identifying a critical damping factor, denoted as $\bar{\beta }=0.2$, which signifies the transition threshold between static and self-rotation states. Beyond this critical value of $\bar{\beta }$, the system’s damping dissipation becomes excessively pronounced to be compensated by the mechanical energy derived from the input thermal energy, consequently stabilizing the turntable system in a static equilibrium state. In contrast, for $\bar{\beta }=0.05$, $\bar{\beta }=0.1$, and $\bar{\beta }=0.15$, the self-rotation can be triggered. Figure 7b displays the effect of the dimensionless damping factor $\bar{\beta }$ on the rotation amplitude and frequency. The larger the damping factor, the more energy loss is generated and, therefore, relatively less energy is available for the system to rotate. Hence, the time required for the system to rotate one revolution increases, indicating that the frequency is decreasing. However, the damping factor has almost no effect on the rotation amplitude.

### 4.4. Effect of Limit Temperature

**Figure 8:**
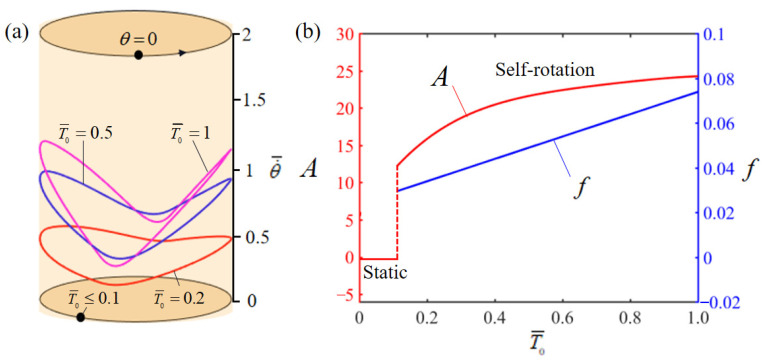
Effect of dimensionless limit temperature ${\bar{T}}_{0}$ on the self-rotation of the LCE-based turntable system, with $\bar{g}=10$, $\theta_{0}=0.5\pi$, $\bar{\alpha }=0.35$, $\bar{\beta }=0.01$, ${\bar{L}}_{0}=1$, ${\bar{k}}_{l}=10$, ${\bar{k}}_{s}=10$, θ=0.25π, and ${\bar{w}}_{0}=1$. (**a**) Limit cycles; (**b**) amplitude and frequency.

Figure 8 illustrates the effect of dimensionless limit temperature ${\bar{T}}_{0}$ on the rotation, in which the other parameters are set to $\bar{g}=10$, $\theta_{0}=0.5\pi$, $\bar{\alpha }=0.35$, $\bar{\beta }=0.01$, ${\bar{L}}_{0}=1$, ${\bar{k}}_{l}=10$, ${\bar{k}}_{s}=10$, θ=0.25π, and ${\bar{w}}_{0}=1$. Figure 8a illustrates the limit cycles of rotation at varying threshold temperatures, revealing a critical threshold temperature of approximately ${\bar{T}}_{0}=0.1$, essential for initiating self-rotation. Below this critical temperature, the energy derived from heat is inadequate to offset the damping losses, leading to the system stabilizing in a static equilibrium state. In contrast, at ${\bar{T}}_{0}=0.2$, ${\bar{T}}_{0}=0.5$, and ${\bar{T}}_{0}=1$, the system demonstrates the capability of self-rotation. Figure 8b presents the influence of the dimensionless threshold temperature ${\bar{T}}_{0}$ on the rotation amplitude and frequency. It is noted that the amplitude escalates in tandem with an increase in the dimensionless threshold temperature. This trend is attributable to the fact that higher threshold temperatures facilitate greater thermal energy generation, resulting in increased energy input to the system and, consequently, an elevated rotation amplitude. And the rotation frequency increases gradually with the increase in limit temperature as well.

### 4.5. Effect of Thermal Shrinkage Coefficient

**Figure 9:**
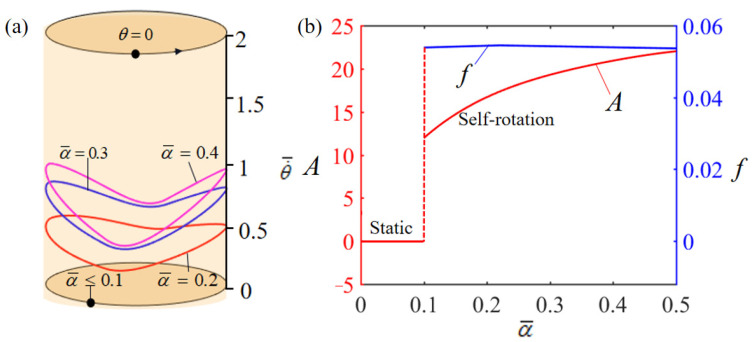
Effect of dimensionless thermal shrinkage coefficient $\bar{\alpha }$ on the self-rotation of the LCE-based turntable system, with ${\bar{T}}_{0}=0.35$, $\theta_{0}=0.5\pi$, $\bar{g}=10$, $\bar{\beta }=0.01$, ${\bar{L}}_{0}=1$, ${\bar{k}}_{l}=10$, ${\bar{k}}_{s}=10$, θ=0.25π, and ${\bar{w}}_{0}=1$. (**a**) Limit cycles; (**b**) amplitude and frequency.

The impact of the dimensionless thermal shrinkage coefficient $\bar{\alpha }$ on rotation behavior is delineated in Figure 9. During the numerical analysis, several parameters were held constant, specified as ${\bar{T}}_{0}=0.35$, $\theta_{0}=0.5\pi$, $\bar{g}=10$, $\bar{\beta }=0.01$, ${\bar{L}}_{0}=1$, ${\bar{k}}_{l}=10$, ${\bar{k}}_{s}=10$, θ=0.25π, and ${\bar{w}}_{0}=1$. Figure 9a illustrates the rotation limit cycles corresponding to various values of $\bar{\alpha }$, revealing a pivotal thermal shrinkage coefficient, approximately $\bar{\alpha }=0.1$, which demarcates the transition from static to self-rotation patterns. For $\bar{\alpha }=0.1$ values below this critical threshold, the system’s thermal energy input is insufficient to counterbalance the energy losses due to damping, leading to a steady-state equilibrium. Conversely, for $\bar{\alpha }=0.2$, $\bar{\alpha }=0.3$, and $\bar{\alpha }=0.4$, the system exhibits self-rotation. Figure 9b demonstrates how the rotation amplitude and frequency are influenced by $\bar{\alpha }$. Notably, the amplitude exhibits a progressive increase with higher values of $\bar{\alpha }$. In contrast, the frequency remains relatively unaffected by changes in the thermal shrinkage coefficient.

### 4.6. Effect of Elastic Stiffness of LCE-Rope

**Figure 10:**
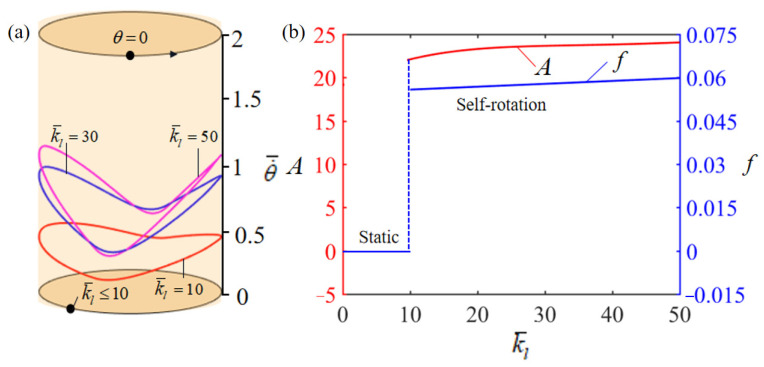
Effect of dimensionless LCE-rope elastic stiffness ${\bar{k}}_{l}$ on the self-rotation of the LCE-based turntable system, with ${\bar{T}}_{0}=0.35$, $\theta_{0}=0.5\pi$, $\bar{\alpha }=0.35$, $\bar{\beta }=0.01$, ${\bar{L}}_{0}=1$, $\bar{g}=10$, ${\bar{k}}_{s}=10$, θ=0.25π, and ${\bar{w}}_{0}=1$. (**a**) Limit cycles; (**b**) amplitude and frequency.

Figure 10a illustrates how the dimensionless elastic stiffness ${\bar{k}}_{l}$ of the LCE-rope influences the rotation. In the numerical calculation, the other parameters are chosen as ${\bar{T}}_{0}=0.35$, $\theta_{0}=0.5\pi$, $\bar{\alpha }=0.35$, $\bar{\beta }=0.01$, ${\bar{L}}_{0}=1$, $\bar{g}=10$, ${\bar{k}}_{s}=10$, θ=0.25π, and ${\bar{w}}_{0}=1$. Figure 10a delineates the limit cycles of rotation for the LCE-rope across a range of dimensionless elastic stiffness values, denoted as ${\bar{k}}_{l}$. A critical threshold for elastic stiffness, identified at ${\bar{k}}_{l}=10$, marks the phase transition from static to self-rotation patterns. When the dimensionless elastic stiffness of the LCE-rope falls below this critical value, the energy converted from heat into the system proves inadequate to counterbalance the energy dissipated through damping effects. This insufficiency results in the turntable ultimately achieving a state of static equilibrium. Instead, for ${\bar{k}}_{l}=10$, ${\bar{k}}_{l}=30$, and ${\bar{k}}_{l}=50$, the system can exhibit self-rotation. Figure 10b depicts how the elastic stiffness of the LCE-rope affects the rotation amplitude and frequency. Only a slight increase in both amplitude and frequency occurs as the elastic stiffness of the LCE-rope is gradually improved. This is attributed to the fact that the elastic stiffness has almost no effect on the deformation of the LCE-rope, and, thus, has a negligible effect on the rotation frequency.

### 4.7. Effect of Elastic Stiffness of Spring

**Figure 11:**
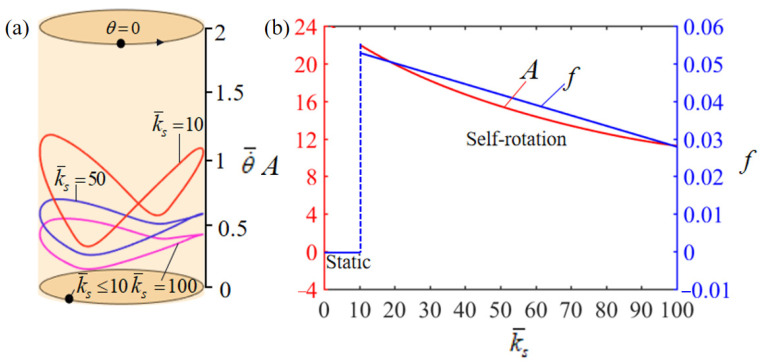
Effect of dimensionless spring elastic stiffness ${\bar{k}}_{s}$ on the self-rotation of the LCE-based turntable system, with ${\bar{T}}_{0}=0.35$, $\theta_{0}=0.5\pi$, $\bar{\alpha }=0.35$, $\bar{\beta }=0.01$, ${\bar{L}}_{0}=1$, ${\bar{k}}_{l}=10$, $\bar{g}=10$, θ=0.25π, and ${\bar{w}}_{0}=1$. (**a**) Limit cycles; (**b**) amplitude and frequency.

Figure 11 shows the effect of the dimensionless elastic stiffness of the spring, ${\bar{k}}_{s}$, on the rotation. In the numerical calculation, we set ${\bar{T}}_{0}=0.35$, $\theta_{0}=0.5\pi$, $\bar{\alpha }=0.35$, $\bar{\beta }=0.01$, ${\bar{L}}_{0}=1$, ${\bar{k}}_{l}=10$, $\bar{g}=10$, θ=0.25π, and ${\bar{w}}_{0}=1$. Figure 11a plots the limit cycles of rotation for different dimensionless elastic stiffnesses of the spring ${\bar{k}}_{s}$. And there presents a critical elastic stiffness of about ${\bar{k}}_{s}=10$ for the phase transition between static and self-rotation patterns. When the elastic stiffness of the spring is below the critical value, the increase in time for the mass ball to return to its initial position within the heat insulation zone results in a decrease in the net work produced by the driving torque, which is insufficient to offset the energy consumed by the damping effect, and the turntable will eventually remain in a static equilibrium position. Conversely, for ${\bar{k}}_{s}=10$, ${\bar{k}}_{s}=50$, and ${\bar{k}}_{s}=100$, the system can undergo self-rotation. Figure 11b illustrates how the elastic stiffness of the spring affects the rotation amplitude and frequency. As the elastic stiffness of the spring is improved, both the amplitude and frequency experience declines. This is due to the fact that an increase in the elastic stiffness of the spring reduces the displacement of the mass ball in the heating zone, thereby diminishing the rotation frequency of the system.

### 4.8. Effect of Heating Zone Angle

**Figure 12:**
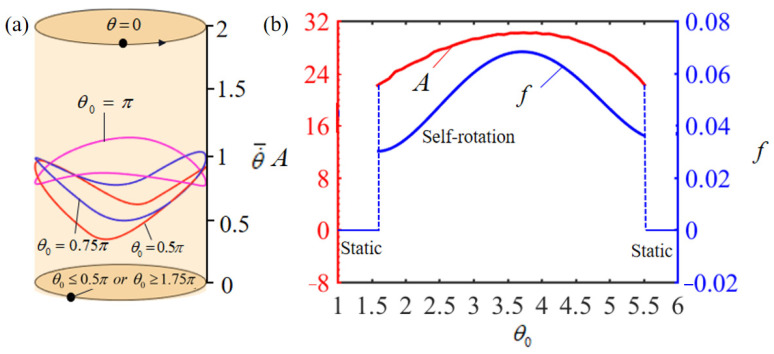
Effect of heating zone angle $\theta_{0}$ on the self-rotation of the LCE-based turntable system, with ${\bar{T}}_{0}=0.35$, $\bar{g}=10$, $\bar{\alpha }=0.35$, $\bar{\beta }=0.01$, ${\bar{L}}_{0}=1$, ${\bar{k}}_{l}=10$, ${\bar{k}}_{s}=10$, θ=0.25π, and ${\bar{w}}_{0}=1$. (**a**) Limit cycles; (**b**) amplitude and frequency.

Figure 12a illustrates the limit cycles of rotation behavior across varying ranges of the heating zone. It identifies two pivotal heating zone angles, approximately $\theta_{0}=0.5\pi$ and $\theta_{0}=1.75\pi$, that demarcate the phase transition from static to self-rotation patterns. For heating zone angles less than the critical threshold of $\theta_{0}=0.5\pi$, the duration of LCE exposure to the heating zone is insufficient. This brevity in exposure results in inadequate thermal energy input to offset the energy losses attributed to damping effects, leading to the turntable maintaining a static equilibrium. Conversely, when the heating zone angle exceeds 1.75π, the LCE-rope’s time in the heat insulation zone is too brief to revert it to its initial state. Consequently, the thermal energy supplied to the system fails to counterbalance the damping dissipation, culminating in the turntable’s persistence in a static equilibrium state. Whereas, for $\theta_{0}=0.5\pi$, $\theta_{0}=0.75\pi$, and $\theta_{0}=\pi$, the system can initiate the self-rotation. The effects of the heating zone angle on the rotation amplitude and frequency are displayed in Figure 12b. As the heating zone angle increases, both the amplitude and frequency present a tendency to increase and then decrease, which is similar to the reason mentioned above that both smaller and larger heating zone ranges negatively affect the rotation of the system.

In summary, the influence of various critical dimensionless parameters on the rotation amplitude and frequency, as explored in this section, is systematically compiled in Table 3. This data offers essential guidance for the engineering of self-excited motion systems, facilitating the precise control of self-excited motion attributes in real-world applications.

## 5. Conclusions

Self-rotating systems exhibit the ability to autonomously harness energy and sustain periodic self-rotation under consistent external stimuli. These systems are increasingly relevant for applications in micro-devices, autonomous robotics, sensors, and energy generation. This study introduces a turntable system based on an electrothermally responsive LCE, capable of self-rotation in a steady-state electrical circuit. By employing an electrothermal LCE model, a nonlinear dynamical model of the system is formulated, and numerical simulations are conducted using the four-order Runge–Kutta method. The simulations reveal two distinct motion regimes for the LCE-based turntable in a steady-state circuit: a static state and a self-rotation state. Critical conditions for initiating self-rotation in a two-mass-ball LCE-based turntable system are quantitatively evaluated, considering various system parameters and their impact on rotation amplitude and frequency. Key parameters influencing self-rotation include gravitational acceleration, initial position of the mass ball, elastic properties of the LCE-rope and spring, limiting temperature, heating zone angle range, thermal shrinkage coefficient, and damping factor. These parameters also govern the system’s rotation frequency and amplitude. These results of this paper are expected to be validated in future experimental works, and the findings of this research offer innovative design perspectives for self-rotating systems, contributing to the understanding of self-rotation principles and broadening potential applications in areas such as energy harvesting, monitoring, soft robotics, medical devices, and micro- and nano-devices. Additionally, these insights provide valuable references for research and development in related disciplines, fostering technological innovation and advancing practical applications. The deployment of self-rotating systems paves the way for more efficient, sustainable, and autonomous energy conversion and utilization, enhancing the scope and efficacy of their use across various domains.

## Figures and Tables

**Table 1:** Material properties and geometric parameters.

Parameter	Definition	Value	Unit
α	Thermal shrinkage coefficient of the LCE material	0–0.5	/
g	Gravitational acceleration	10	${m/s}^{2}$
β	Damping factor	0.001~0.01	kg/s
$\theta_{0}$	Heating zone angle	0.4π~0.8π	/
θ	Initial angle of the mass ball	0~2π	/
$w_{0}$	Initial angular velocity	0.4~2	rad/s
$k_{s}$	Elastic stiffness of the spring	0.005~50	N/m
$k_{l}$	Elastic stiffness of the LCE-rope	0.005~50	N/m
$L_{0}$	Distance from mass ball to turntable center	0.04~0.16	m
$L_{s}$	Initial length of spring	1.6~20	mm
$L_{i}$	Initial length of LCE-rope	0.02	m
τ	Characteristic time of heat exchange between LCE-rope and the environment	0.001~0.1	s
m	Mass of small mass ball	0.01	kg
$T_{0}$	Limit temperature difference of LCE-rope	0–20	℃
$\rho_{c}$	Specific heat capacity of LCE material	1000~4500	$J\;/\left(\mathrm{kg}℃\right)$
q	Heat generated by the thermal effect of electric current	0~10	J /s

**Table 2:** Dimensionless parameters.

Parameter	${\bar{T}}_{0}$	$\bar{g}$	$\bar{\beta }$	${\bar{L}}_{0}$	$\bar{\alpha }$	${\bar{k}}_{l}$	${\bar{k}}_{s}$	$\theta_{0}$
Value	0.1~0.5	6~20	0.01~0.2	0.5~3	0.38~0.45	10~100	10~100	0.5π~1.5π

**Table 3:** Effects of several key dimensionless parameters.

Dimensionless Parameter	Amplitude	Frequency
$\bar{g}$	increases with increasing $\bar{g}$	increases with increasing $\bar{g}$
${\bar{L}}_{0}$	decreases with increasing ${\bar{L}}_{0}$	decreases with increasing ${\bar{L}}_{0}$
$\bar{\beta }$	not affected by $\bar{\beta }$	decreases with increasing $\bar{\beta }$
${\bar{T}}_{0}$	increases with increasing ${\bar{T}}_{0}$	increases with increasing ${\bar{T}}_{0}$
$\bar{\alpha }$	increases with increasing $\bar{\alpha }$	not affected by $\bar{\alpha }$
${\bar{k}}_{l}$	increases slightly with increasing ${\bar{k}}_{l}$	increases slightly with increasing ${\bar{k}}_{l}$
${\bar{k}}_{s}$	decreases with increasing ${\bar{k}}_{s}$	decreases with increasing ${\bar{k}}_{s}$
$\theta_{0}$	increases and then decreases with increasing $\theta_{0}$	increases and then decreases with increasing $\theta_{0}$

## Data Availability

The data that support the findings of this study are available upon reasonable request from the authors.
